# From multiallele fish to nonstandard environments, how ZFIN assigns phenotypes, human disease models, and gene expression annotations to genes

**DOI:** 10.1093/genetics/iyad032

**Published:** 2023-03-02

**Authors:** Yvonne M Bradford, Ceri E Van Slyke, Douglas G Howe, David Fashena, Ken Frazer, Ryan Martin, Holly Paddock, Christian Pich, Sridhar Ramachandran, Leyla Ruzicka, Amy Singer, Ryan Taylor, Wei-Chia Tseng, Monte Westerfield

**Affiliations:** The Institute of Neuroscience, University of Oregon, Eugene, OR 97403-1254, USA; The Institute of Neuroscience, University of Oregon, Eugene, OR 97403-1254, USA; The Institute of Neuroscience, University of Oregon, Eugene, OR 97403-1254, USA; The Institute of Neuroscience, University of Oregon, Eugene, OR 97403-1254, USA; The Institute of Neuroscience, University of Oregon, Eugene, OR 97403-1254, USA; The Institute of Neuroscience, University of Oregon, Eugene, OR 97403-1254, USA; The Institute of Neuroscience, University of Oregon, Eugene, OR 97403-1254, USA; The Institute of Neuroscience, University of Oregon, Eugene, OR 97403-1254, USA; The Institute of Neuroscience, University of Oregon, Eugene, OR 97403-1254, USA; The Institute of Neuroscience, University of Oregon, Eugene, OR 97403-1254, USA; The Institute of Neuroscience, University of Oregon, Eugene, OR 97403-1254, USA; The Institute of Neuroscience, University of Oregon, Eugene, OR 97403-1254, USA; The Institute of Neuroscience, University of Oregon, Eugene, OR 97403-1254, USA; The Institute of Neuroscience, University of Oregon, Eugene, OR 97403-1254, USA

**Keywords:** zebrafish, *Danio rerio*, phenotype, human disease model, expression, ZFIN, model organism database

## Abstract

*Danio rerio* is a model organism used to investigate vertebrate development. Manipulation of the zebrafish genome and resultant gene products by mutation or targeted knockdown has made the zebrafish a good system for investigating gene function, providing a resource to investigate genetic contributors to phenotype and human disease. Phenotypic outcomes can be the result of gene mutation, targeted knockdown of gene products, manipulation of experimental conditions, or any combination thereof. Zebrafish have been used in various genetic and chemical screens to identify genetic and environmental contributors to phenotype and disease outcomes. The Zebrafish Information Network (ZFIN, zfin.org) is the central repository for genetic, genomic, and phenotypic data that result from research using *D. rerio*. Here we describe how ZFIN annotates phenotype, expression, and disease model data across various experimental designs, how we computationally determine wild-type gene expression, the phenotypic gene, and how these results allow us to propagate gene expression, phenotype, and disease model data to the correct gene, or gene related entity.

## Introduction

Understanding gene and protein function can provide insight to elucidate the intricate cellular mechanisms that are responsible for the development, growth, pathology, and senescence of organisms. Observing the results of gene mutations is the cornerstone of elucidating and understanding gene function. The zebrafish, *Danio rerio*, has been used in forward and reverse genetic screens to study gene function and understand the mechanisms of vertebrate development (Driever *et al.* 1996; Haffter *et al.* 1996; Golling *et al.* 2002; Moens *et al.* 2008; Varshney *et al.* 2013). The results of gene function studies in zebrafish are relevant to understanding human gene function due to the conservation of gene sequences and functions between zebrafish and humans (Postlethwait *et al.* 2000; Howe, Clark *et al.* 2013). Due to similarities between zebrafish and human organ functions and physiology, zebrafish have been used to model human diseases that affect the cardiovascular (Smith *et al.* 2009; Liu *et al.* 2019), nervous (Chapman *et al.* 2013; Hin *et al.* 2020), visual (Zhang *et al.* 2016), muscular (Majczenko *et al.* 2012; Widrick *et al.* 2016), and many other systems. In addition to understanding gene function and disease pathogenesis, zebrafish are increasingly used for toxicology and drug discovery studies, as well as research that explores the effects of genotype and environment on phenotype and disease (Zon and Peterson 2005; Kaufman *et al.* 2009; Williams *et al.* 2014; Wheeler *et al.* 2019; Cassar *et al.* 2020).

The Zebrafish Information Network, ZFIN (zfin.org), is the database resource for zebrafish research that annotates, curates, and makes data available from zebrafish research that spans genetic perturbations, chemically induced phenotypes, and human disease models, as well as gene expression (Sprague *et al.* 2008; Ruzicka *et al.* 2015; Howe *et al.* 2017). ZFIN curates gene expression, phenotype, and human disease model data by annotating the genotypes, experimental conditions, anatomical structures, phenotype statements, and disease models reported in zebrafish research publications (Sprague *et al.* 2006; Howe, Bradford *et al.* 2013; Bradford *et al.* 2017). These annotations can include genotypes with one or many alleles and experimental conditions that range from standard conditions to manipulation of temperature, diet, chemicals, or other conditions. Due to the breadth of data that represent combinations of genotype and environment that produce a phenotypic outcome or human disease model, it can be challenging to determine whether a particular allele or environment is causative. To understand gene function and clarify how gene dysfunction contributes to disease, it is necessary to separate genetic phenotypes from those caused by the environment. ZFIN has developed a data model and algorithms that distinguish the genotype and environment components of an annotation to parse genetic and environmental contributors to phenotypes, using the results to infer which genes are causative of a phenotype. Here we discuss the ZFIN annotation components and computational logic used to infer wild-type gene expression, gene-phenotype and gene-human disease relationships, and the ZFIN webpages and download files (https://zfin.org/downloads) where the data are available.

## ZFIN annotation components

There are three main components to ZFIN gene expression, phenotype, and human disease model annotations: (1) the genotype of the fish including gene knockdown reagents used, (2) the experimental conditions applied, and (3) an ontological representation of the results.

### Fish

Gene mutation and sequence targeting reagents (STRs), which knockdown gene products, are routinely used in zebrafish to study gene function. To represent all of the genes that are affected due to either gene mutation or knockdown, ZFIN uses a data model that groups the genotype and applied STR in an object called Fish. Mutant gene loci are curated as alleles of genes and are part of a genotype together with the background strain when that information is provided. Zebrafish are also amenable to transgene insertion to knock out genes (Amsterdam *et al.* 2004), overexpress endogenous or other species genes (Sabaawy *et al.* 2006; Padanad *et al.* 2012), insert mutant genes (Kimelman *et al.* 2017; Endo *et al.* 2022), or express fluorescent proteins to mark anatomical structures (Lawson and Weinstein 2002; Clark *et al.* 2011). Transgene insertion is accomplished by the injection of DNA constructs (transgenic constructs) into zebrafish embryos, which are then raised to maturity and screened for stable germline transmission (Stuart *et al.* 1990; Culp *et al.* 1991). ZFIN creates records for transgenic constructs and makes an association with the transgenic genomic features (alleles) using a phenotypic or innocuous relationship. The phenotypic relationship is used with constructs that drive expression of either an endogenous zebrafish gene or a gene from another species (Table 1). These constructs are expected to produce protein products that can have a phenotypic effect. The innocuous relationship is used with constructs that drive the expression of fluorescent proteins or are unable to transcribe a protein product unless inserted near a native promoter, such as gene trap constructs. Information on the innocuous or phenotypic relationship between a genomic feature and a construct is available in the “Innocuous/phenotypic construct details” download file. Transgenic alleles are represented in the genotype when applicable, and genotypes are considered innocuous or phenotypic depending on the relationship between the allele and construct. Site-specific mutagenesis using CRISPRs and TALENs is also used in zebrafish to screen for candidate genes (Jao *et al.* 2013; Zu *et al.* 2013). Zebrafish crispants, F0 founder zebrafish created using CRISPRs, are also used to phenocopy loss of function mutants (Bek *et al.* 2021). In addition, gene function can be investigated in zebrafish using morpholinos, which knockdown the gene by targeting RNA, effectively silencing the gene product (Nasevicius and Ekker 2000; Ekker and Larson 2001). ZFIN group morpholinos, CRISPRs, and TALENs in a class called STR due to the sequence-specific nature of these reagents. Both alleles and STRs have relationships with the genes they knockout or target. ZFIN developed the Fish data model to facilitate the identification of causative genes due to the many ways in which gene function is investigated in zebrafish.

**Table 1.** Innocuous and phenotypic constructs.

Genomic feature	Relationship	Construct	Construct description
rw021Tg	Contains innocuous sequence feature	Tg(atoh7:GFP)	Promoter for *atoh7* drives expression of GFP
ncu102Tg	Contains innocuous sequence feature	Tg(hsp70l:cyfip2_C179R-EGFP)	Promoter for *hsp70l* drives mutant *cyfip2* that produces protein change of C to R at position 179
ua3162Tg	Contains phenotypic sequence feature	Tg(opn1sw1:nrl)	Promoter for *opn1sw1* drives expression of *nrl*
ns103Tg	Contains phenotypic sequence feature	Tg(rag2:Hsa.ALDH1A2)	Promoter for *rag2* drives expression of Human gene ALD1A2 expression

### Experimental conditions

Zebrafish are used in a wide array of experimental contexts. To represent the experiments reported in research publications, the conditions applied are curated using ontology terms from the Zebrafish Experimental Conditions Ontology (ZECO; Bradford *et al.* 2016) along with terms from the Zebrafish Anatomy Ontology (ZFA; Van Slyke *et al.* 2014), Gene Ontology Cellular Component (GO-CC; Ashburner *et al.* 2000; Carbon *et al.* 2019), Chemical Entities of Biological Interest (ChEBI; Hastings *et al.* 2016), and NCBI Taxon (Federhen 2012). The ZECO ontology contains the main types of conditions with high-level nodes that include standard conditions for zebrafish husbandry as described in The Zebrafish Book (Westerfield 2000), control conditions (such as vehicle injections), biological treatment (such as exposure to bacteria), chemical treatment, diet alterations, housing conditions, in vitro culture, surgical manipulation, lighting conditions, temperature exposure, radiation exposure, and water quality. ZECO terms from the biological treatment branch are combined with NCBI Taxon terms to annotate conditions where another organism is added to the environment or when the zebrafish are raised in germ-free environments. The chemical treatment branch of ZECO is combined with chemicals from the ChEBI ontology to annotate the chemical that was used in the experiment. The surgical manipulation branch is combined with terms from the ZFA ontology to denote the anatomical structures that underwent ablation, resections, or other surgical manipulations. In instances when a cellular component, such as an axon, is ablated, GO-CC terms are used along with ZFA terms.

### Ontological representation of results

ZFIN uses multiple ontologies to annotate gene expression, phenotype, and human disease models. Disease, expression, and phenotype annotations include the Fish and experimental conditions. To complete disease annotations, terms from the Disease Ontology (DO; Schriml *et al.* 2019) are added as well as evidence terms from the Evidence and Conclusion Ontology (ECO; Nadendla *et al.* 2022). To describe the location of the expression or phenotype annotation, terms from the ZFA, the Zebrafish Stage Ontology (ZFS; Van Slyke *et al.* 2014), GO-CC, and Spatial Ontology (BSPO; Dahdul *et al.* 2014) are used. Expression annotations include the gene that is expressed as well as the assay type using terms from the Measurement Method Ontology (Smith *et al.* 2013). Annotations that describe the phenotypes of biological metabolites use ChEBI terms and those pertaining to the biological process or molecular function of a gene use GO Molecular Function (GO-MF) or GO Biological Process (GO-BP) terms. All phenotype annotations use terms from the Phenotype and Trait Ontology (PATO; Gkoutos *et al.* 2005) as well as tags for “normal,” “abnormal,” “ameliorated,” or “exacerbated.” Phenotype annotations that use terms from GO-BP or GO-MF only use PATO terms from the process quality branch, while anatomical entity phenotype annotations use terms from the physical object quality branch. All ZFIN annotations refer to the publication that reported the results.

In summary, ZFIN gene expression, phenotype, and disease model annotations are multipartite, including the genotype and applied knockdown reagents as Fish, the experimental conditions, and the ontological representation of the results. See Tables 2–4 for examples of gene expression, phenotype, and human disease model annotations.

**Table 2.** Gene expression annotations.

Gene	Fish	Experimental Condition	Stage	Expression	Reference
*pax2a*	AB	Standard conditions [ZECO:0000103]	Pharyngula: Prim-25 [ZFS:0000031]	Optic furrow [ZFA:0005491]	ZDB-PUB-180407-9; PMID: 29625437
*pax2a*	*aldh1a* ^i26/i26^	Standard conditions [ZECO:0000103]	Segmentation: 10–13 somites [ZFS:0000025]	Lateral plate mesoderm [ZFA:0000121]	ZDB-PUB-011002-4; PMID: 11688558
*pax2a*	*cyp26a1* ^rw716/rw716^	Chemical treatment: all-trans-retinoic acid [ZECO:0000111], [CHEBI:15367]	Segmentation: 1–4 somites [ZFS:0000023]	Midbrain hindbrain boundary neural keel [ZFA:0007045]	ZDB-PUB-061227-41; PMID: 17164423
*pax2a*	AB + MO6-pax8 + MO7-pax8	Standard conditions [ZECO:0000103]	Segmentations: 5–9 somites [ZFS:0000024]	Epibranchial field [ZFA:0007061]	ZDB-PUB-110119-6; PMID: 21215261

**Table 3.** Phenotype annotations.

Fish	Experimental conditions	Stage	Phenotype	Reference
*sox9a* ^tw37/tw37^	Standard conditions [ZECO:0000103]	Larval: Day 5 [ZFS:0000037]	Ceratohyal cartilage decreased size, abnormal [ZFA:0001400], [PATO:0000587]	ZDB-PUB-970210-30; PMID: 9007254
hu11688Tg + MO1-tnnt2a(TL)	Chemical treatment by environment: isoprenaline [ZECO:0000238], [CHEBI:64317]	Larval: Protruding-mouth [ZFS:0000035]	Heart contraction increased rate, abnormal [GO:0060047], [PATO:0000912]	ZDB-PUB-181004-5; PMID: 30279735
AB + CRISPR1-cyp1b1 + CRISPR2-cyp1b1	Standard Conditions [ZECO:0000103]	Larval: Day 6 [ZFS:0000038]	Ventral mandibular arch immature, abnormal [ZFA:0001273], [PATO:0001501]	ZDB-PUB-210703-31; PMID: 34208498
x17Tg	Heat shock [ZECO:0000166]	Larval: Protruding-mouth [ZFS:0000035]	Posterior macula mislocalized, abnormal [ZFA:0000558], [PATO:0000628]	ZDB-PUB-190426-5; PMID: 31022185
AB	Chemical treatment by diet: resveratrol [ZECO:0000239], [CHEBI:27881]	Adult [ZFS:0000044]	Blood triglyceride decreased amount, abnormal [ZFA:0000007], [CHEBI:17855], [PATO:0001997]	ZDB-PUB-170708-6; PMID: 28686680

**Table 4.** Human disease model annotations.

Fish	Experimental conditions	Human disease	Reference
*rps19* ^zf556/zf5556^	Standard conditions [ZECO:0000103]	Diamond-Blackfan anemia [DOID:1339]	ZDB-PUB-140728-17; PMID: 25058426
WT + MO1-rpl11	Standard conditions [ZECO:0000103]	Diamond-Blackfan anemia [DOID:1339]	ZDB-PUB-151021-8; PMID: 26484089
WT	Chemical treatment: pentetrazol [ZECO:0000111], [CHEBI:34910]	Epilepsy [DOID:1826]	ZDB-PUB-160311-7; PMID: 26961169
AB	Fungal treatment by injection: *Candida albicans* [ZECO:0000232], [NCBITaxon:5476]	Candidiasis [DOID:1508]	ZDB-PUB-200119-2; PMID: 31952292

## Database logic for gene expression, gene-phenotype, and gene-disease associations

As described in the previous section, each data type provides different information used to construct an annotation. To be able to understand the function of a single gene, it is necessary to isolate the environmental factors from the genetic interactions within an annotation and ensure correct attribution of the experimental outcome to a single gene, if appropriate. To ensure the correct representation of data sets and data displays on the gene page, ZFIN has established query logic or algorithms to parse the details of existing annotations such that the gene page only displays those data that show where a gene is normally expressed and the phenotypic results of mutation or knockdown of that specific gene, as explained in the sections below.

### Wild-type gene expression

**Fig. 1.**
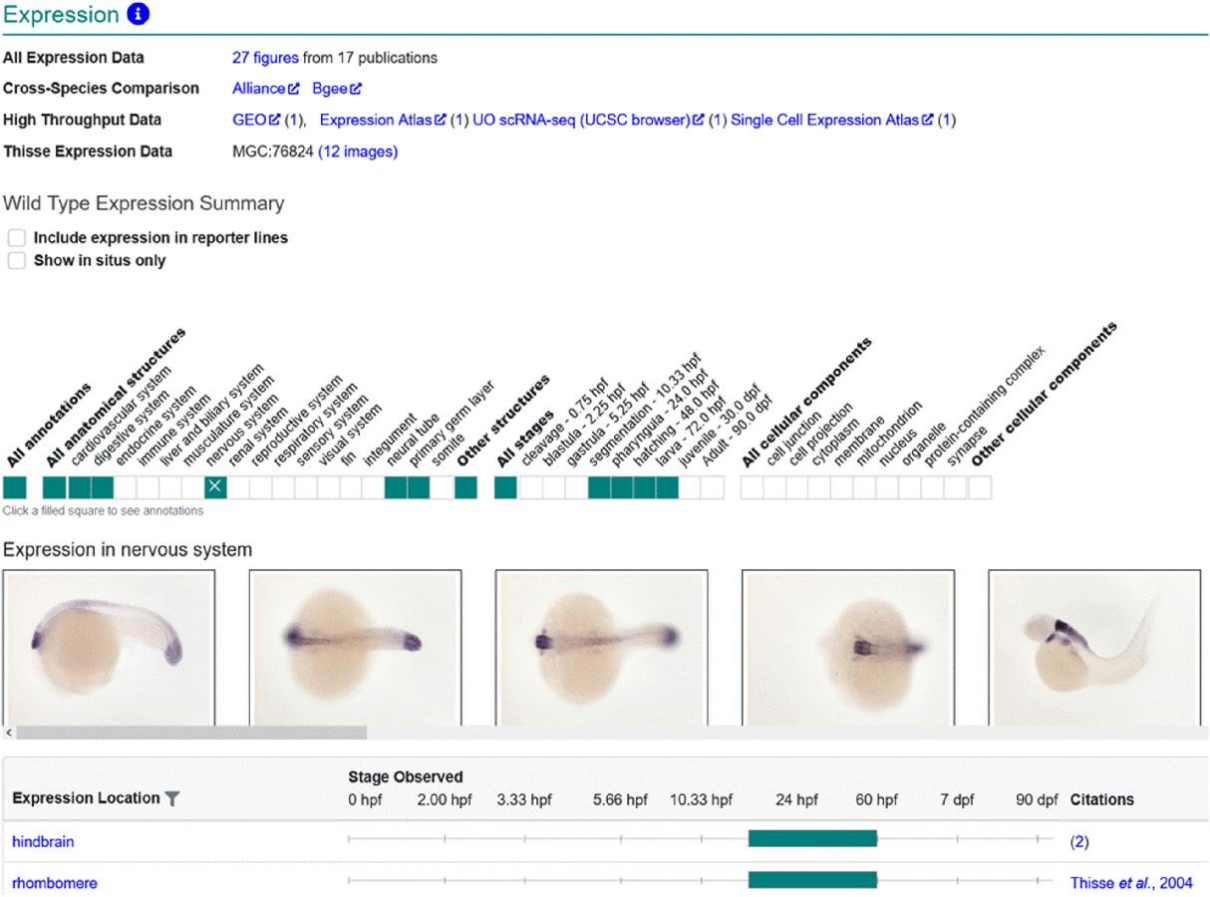
Gene page gene expression. Gene expression displayed on the gene page is limited to gene expression results in wild-type backgrounds. The Wild-Type Expression Summary displays a graphical ribbon that denotes the anatomical systems and stages that have gene expression annotations. The table lists the anatomical terms, stages, and citations.

Understanding the wild-type expression profile of genes is essential to understand what systems and structures a gene contributes to developmentally and is necessary as a comparator when evaluating gene expression in mutant or gene-knockdown zebrafish. ZFIN curators annotate gene expression in both wild-type and mutant backgrounds as well as what experimental conditions are present. To determine wild-type gene expression, algorithms are designed to identify gene expression in Fish that have wild-type backgrounds, no mutant alleles, in standard or control conditions. Gene expression results that meet these criteria are displayed on the gene page (Fig. 1) and are provided in the “Expression data for wild-type fish” download file available on the downloads page. ZFIN also provides wild-type gene expression annotations to the Alliance of Genome Resources (Alliance, www.alliancegenome.org; (Agapite *et al.* 2022). Mutant or non-wild-type zebrafish gene expression can be found on the Fish page, via the search interface, in the download file “ZFIN genes with expression assay records,” and on STR pages. The STR page displays expression only in Fish where a single STR is used in a wild-type background, highlighting the effects of the individual STR on gene expression (Fig. 4).

### Affected genes for phenotype and disease models

**Fig. 2.**
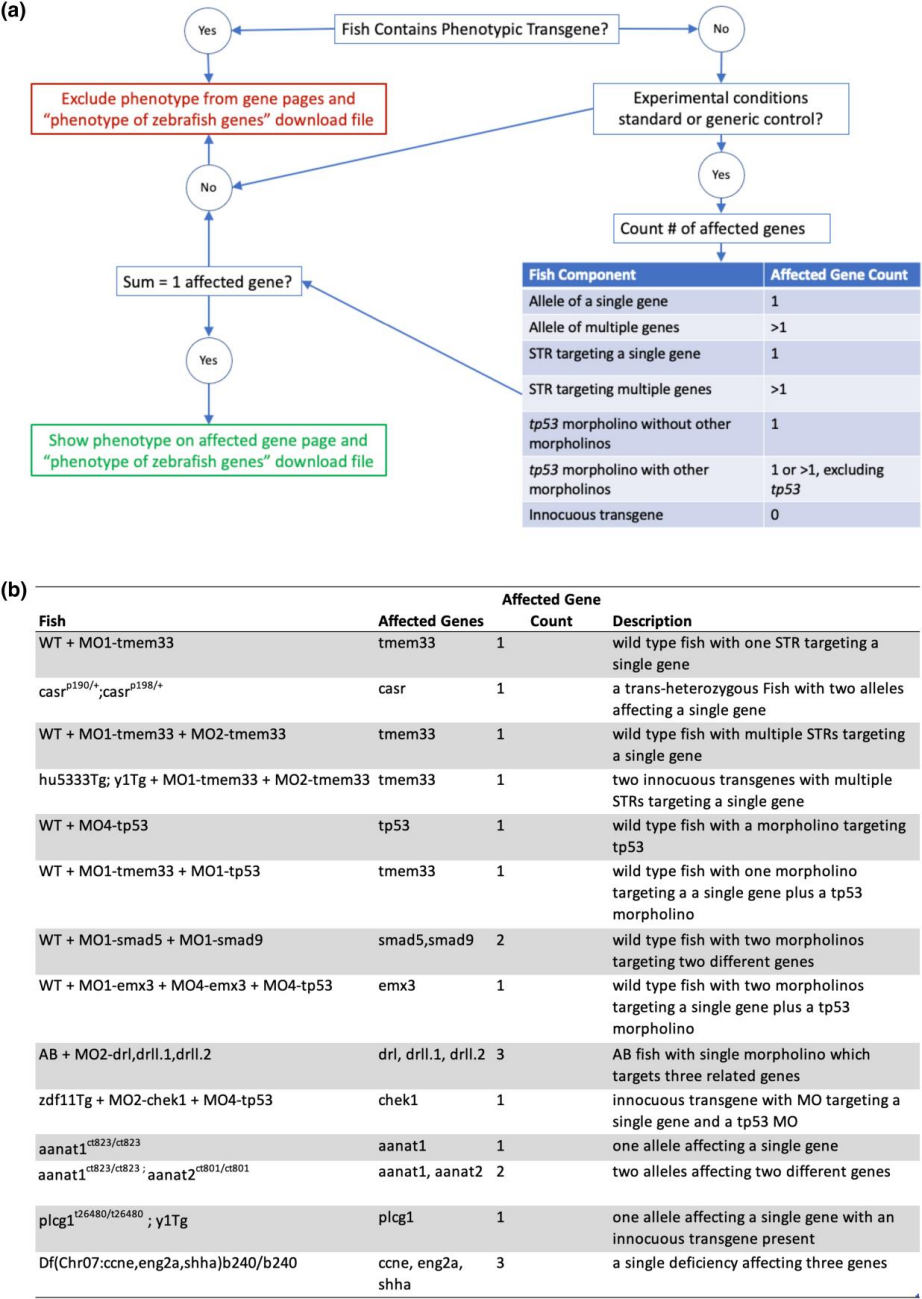
Logic for determining Fish affected gene count. a) A logic flow diagram describing the algorithm used to determine number of affected genes in a Fish and whether phenotype data can be shown on a gene page. b) A table of examples of Fish that result in variable numbers of affected genes.

To determine the function of a gene, it is instructive to look at the phenotypic outcomes of mutant and gene-knockdown zebrafish. Phenotype can encompass many levels of observation from morphologic changes at the level of the whole organism to changes in gene expression and protein location within a cell. To draw conclusions about what functions a gene has in the cell or organism, it is necessary to ensure that the phenotypes attributed to the gene are solely caused by changes to that gene. ZFIN has developed algorithms to determine the total number of altered or affected genes in a Fish, with the resulting number determining if a causative gene can be inferred. The number of affected genes is determined by counting distinct genes associated with alleles and STRs that are associated with a Fish. When the affected gene count equals one and the experimental conditions are standard/generic control, the phenotype or disease association is inferred or calculated to be caused by the gene associated with the Fish either by its allele relationship or by its STR target relationship. There are various ways to arrive at gene count equals one. As illustrated in Fig. 2, Fish can have one affected gene but can be more or less complex in their genetic makeup. For example, a Fish with a single allele with one affected gene, a Fish with multiple alleles where all alleles affect the same gene, a wild-type Fish injected with one or more STRs targeting one gene, and a nonphenotypic transgenic line injected with one or more STRs targeting one gene all have only a single affected gene.

**Fig. 3.**
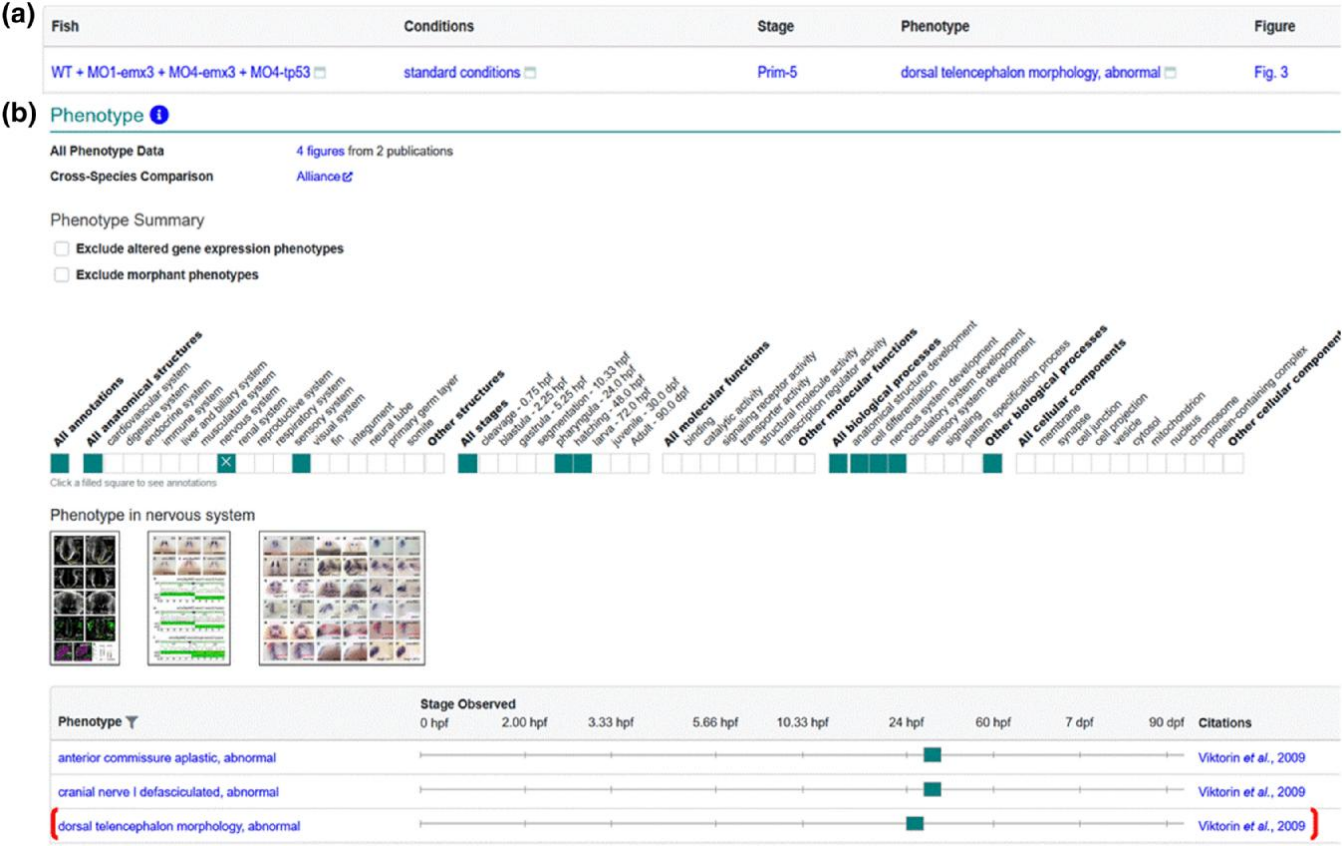
Display of MO-*tp53* Fish data on gene page. a) Phenotype data for Fish WT + MO1-emx3 + MO4-emx3 + MO4-tp53 in standard conditions as reported in Viktorin *et al.* (2009). b) The phenotype summary section on the *emx3* gene page has a ribbon that denotes systems, stages, biological processes, and cellular components that have annotations, with individual annotations displayed in the table. Thumbnail images are displayed when available. Phenotype corresponding to Fish in A is denoted by bracket.

We have recently added rules to the algorithm that do not count *tp53* as an affected gene in Fish, where morpholinos against *tp53* were used in addition to non-tp53 morpholinos due to the way zebrafish researchers use morpholinos against tp53 to deal with nonspecific effects (Robu *et al.* 2007). Previously, a Fish that had two morpholinos, one of which was against *tp53*, would be considered to have two affected genes, and the phenotype would be excluded from gene pages. The algorithm now ignores *tp53* morpholinos in the Fish and the resulting group of morpholinos is used to obtain the affected gene count, with data propagated to the gene page when the gene count equals one (Fig. 3).

In addition to counting the number of affected genes, the algorithms account for transgenic lines, both those that are treated as wild-type equivalents by the research community and those used to alter the expression of a gene. As explained in the previous Fish section, Fish containing genomic features that have a phenotypic relationship to a construct are considered phenotypic lines. These Fish are excluded by affected gene count algorithms because phenotype and disease annotations using such Fish cannot be attributed to a single gene. This is due to the lack of gene counting for genes expressed by transgenic constructs, as the algorithm does not count the genes associated with constructs, instead it solely relies on the phenotypic relationship between transgenic allele and construct. Since the algorithm does not count genes associated with transgenic constructs, it is unable to identify the number of genes a construct has. Fish that have genomic features with an innocuous relationship to a construct are considered innocuous and are counted as wild-type equivalents by the affected gene count algorithms. The resulting data allow us to determine computationally the affected gene count. In addition to gene count and innocuous or phenotypic genomic features, the experimental conditions are also taken into account when determining whether the phenotype or disease model data can be attributed to a gene. When the experimental conditions are standard or generic control and the affected gene count is one, the resulting phenotype or disease association is inferred to be caused by the one affected gene. These data are then propagated to the gene page, gene-related entity pages, and download files. Currently, only phenotype annotations that are tagged as “abnormal” are displayed in the phenotype section of gene pages, as those annotations directly relate to individual gene functions. Phenotype statements that are tagged, “ameliorated,” or “exacerbated” are usually the result of genetic interactions or applied experimental conditions and do not conform to the single affected gene algorithm. Ameliorated and exacerbated annotations are displayed on the Fish page, can be found via the search interface, and in “Ameliorated phenotypes” and “Exacerbated phenotype” download files.

**Fig. 4.**
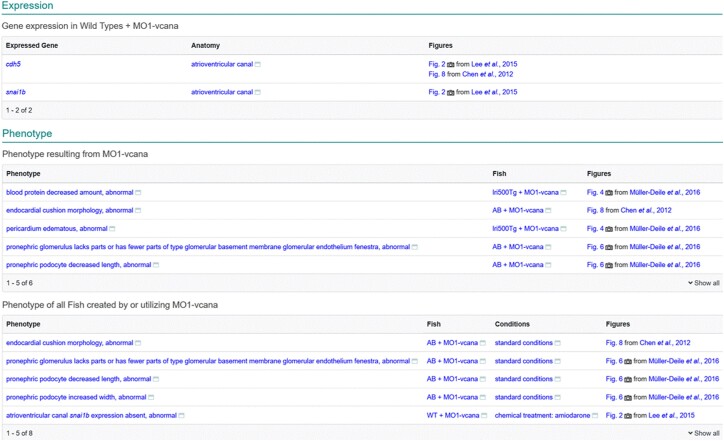
STR page. Expression display is limited to Fish with a wild-type background under standard or control conditions. Phenotype display is divided into two sections, the first labeled “Phenotype resulting from MO1-vcana” contains phenotype only in wild-type or innocuous transgenic fish with standard conditions. Phenotype in more complex fish or under nonstandard conditions as well as the phenotype from the previous section is displayed in the section labeled “Phenotype of all Fish created by or utilizing MO1-vcana.”

Similar rules are employed for determining whether a phenotype is caused by an STR or may be the result of a combination of genetic affectors. On the STR page, phenotype in Fish with only a single STR targeting a single gene in a wild-type or nonphenotypic transgenic background is displayed in the section where the label starts with “Phenotype resulting from” followed by the STR name (Fig. 4). For more complex Fish or when the STR has multiple targets, the phenotypes are displayed in a section labeled “Phenotype of all Fish created by or utilizing” followed by the STR name(s).

**Fig. 5.**
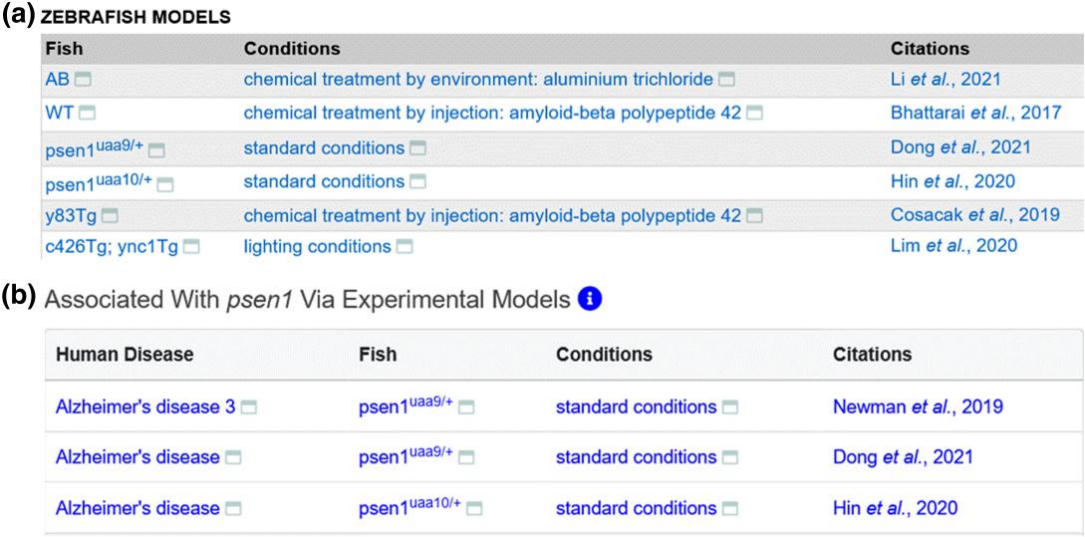
Display of disease model data. a) Zebrafish Models table from the Alzheimer's disease term page displaying all Fish and experimental conditions that are annotated as disease models. b) Human disease model table from the *psen1* gene page, showing the diseases associated with *psen1* via experimental models that have a single affected gene Fish in standard conditions.

The algorithm for determining the number of affected genes in Fish for phenotype displays is also used to display disease model data on a gene page. Zebrafish models of human disease can be either genetic models or models induced by experimental conditions or a combination of these (Kawahara *et al.* 2011; Cronin and Grealy 2017; Yu *et al.* 2021). ZFIN curators make disease model annotations when research publications report zebrafish models of human diseases. Zebrafish disease model annotations contain Fish, experimental conditions, disease terms, ECO evidence codes, and references. All annotated zebrafish models of a disease are displayed on ZFIN disease term pages (Fig. 5a). Disease models that have a Fish with a single affected gene with standard or control experimental conditions are displayed on the corresponding gene page in the human disease model table (Fig. 5b). ZFIN does not annotate when a Fish is *not* a model of a human disease, as this is not usually reported in the literature. Zebrafish models of human disease data are provided in the “Human disease models” download file. In addition, ZFIN provides phenotype and disease model data to the Alliance.

### Conclusion

The development, growth, and senescence of organisms are the result of an elegant orchestra of gene expression, protein function, pathology, and the environment. Understanding gene and protein function is essential knowledge that provides insight into the cellular mechanisms of developmental and disease processes. Gene function has traditionally been elucidated using gene mutation and targeted gene knockdown. Genetic and experimental condition manipulation, either singly or in combination, produces phenotypic outcomes. Zebrafish have been used in forward and reverse genetic screens to study gene function, model human disease, understand toxicology, and discover drugs. ZFIN curates genetic, genomic, phenotypic, and disease model data that result from zebrafish research. The algorithms used by ZFIN support the identification of wild-type expression patterns, genes that are causative for phenotypes, and disease models from data collected in a wide variety of Fish and experimental conditions. The resulting data are presented on the gene, STR, and disease pages as well as in specialized download files. The aggregation of these data on discrete pages and download files allows users to quickly synthesize data about gene function, phenotypic outcomes, and disease models without having to manually compile the research from many genotypes, gene knockdowns, and experimental conditions.

## Data Availability

All relevant data are available at ZFIN, zfin.org.

## References

[iyad032-B1] Agapite J , Albou LP, Aleksander SA, Alexander M, Anagnostopoulos AV, Antonazzo G, Argasinska J, Arnaboldi V, Attrill H, Becerra A, et al Harmonizing model organism data in the alliance of genome resources. Genetics 2022;220(4):iyac022. doi:10.1093/GENETICS/IYAC022. 35380658 PMC8982023

[iyad032-B2] Amsterdam A , Nissen RM, Sun Z, Swindell EC, Farrington S, Hopkins N. Identification of 315 genes essential for early zebrafish development. Proc Natl Acad Sci U S A. 2004;101(35):12792–12797. doi:10.1073/PNAS.0403929101. 15256591 PMC516474

[iyad032-B3] Ashburner M , Ball CA, Blake JA, Botstein D, Butler H, Cherry JM, Davis AP, Dolinski K, Dwight SS, Eppig JT, et al Gene ontology: tool for the unification of biology. Nat Genet. 2000;25(1):25–29. doi:10.1038/75556. 10802651 PMC3037419

[iyad032-B4] Bek JW , Shochat C, De Clercq A, De Saffel H, Boel A, Metz J, Rodenburg F, Karasik D, Willaert A, Coucke PJ. Lrp5 mutant and crispant zebrafish faithfully model human osteoporosis, establishing the zebrafish as a platform for CRISPR-based functional screening of osteoporosis candidate genes. J Bone Miner Res. 2021;36(9):1749–1764. doi:10.1002/JBMR.4327. 33957005

[iyad032-B5] Bradford YM , Toro S, Ramachandran S, Ruzicka L, Howe DG, Eagle A, Kalita P, Martin R, Moxon SAT, Schaper K, et al Zebrafish models of human disease: gaining insight into human disease at ZFIN. ILAR J. 2017;58(1):4–16. doi:10.1093/ilar/ilw040. 28838067 PMC5886338

[iyad032-B6] Bradford YM , Van Slyke CE, Toro S, Ramachandran S. The zebrafish experimental conditions ontology: systemizing experimental descriptions in ZFIN. In: CEUR Workshop Proceedings, 1747; 2016. Available from: http://ceur-ws. org/ Vol- 1747/ IP25_ ICBO2 016. pdf.

[iyad032-B7] Carbon S , Douglass E, Dunn N, Good B, Harris NL, Lewis SE, Mungall CJ, Basu S, Chisholm RL, Dodson RJ, et al The gene ontology resource: 20 years and still GOing strong. Nucleic Acids Res. 2019;47(D1):D330–D338. doi:10.1093/nar/gky1055. 30395331 PMC6323945

[iyad032-B8] Cassar S , Adatto I, Freeman JL, Gamse JT, Iturria I, Lawrence C, Muriana A, Peterson RT, Van Cruchten S, Zon LI. Use of zebrafish in drug discovery toxicology. Chem Res Toxicol. 2020;33(1):95–118. doi:10.1021/acs.chemrestox.9b00335. 31625720 PMC7162671

[iyad032-B9] Chapman AL , Bennett EJ, Ramesh TM, De Vos KJ, Grierson AJ. Axonal transport defects in a Mitofusin 2 loss of function model of Charcot-Marie-tooth disease in zebrafish. PLoS One. 2013;8(6):e67276. doi:10.1371/JOURNAL.PONE.0067276. 23840650 PMC3694133

[iyad032-B10] Clark BS , Winter M, Cohen AR, Link BA. Generation of Rab-based transgenic lines for in vivo studies of endosome biology in zebrafish. Dev Dyn. 2011;240(11):2452–2465. doi:10.1002/DVDY.22758. 21976318 PMC3197968

[iyad032-B11] Cronin A , Grealy M. Neuroprotective and neuro-restorative effects of minocycline and rasagiline in a zebrafish 6-hydroxydopamine model of Parkinson's disease. Neuroscience 2017;367:34–46. doi:10.1016/j.neuroscience.2017.10.018. 29079063

[iyad032-B12] Culp P , Nüsslein-Volhard C, Hopkins N. High-frequency germ-line transmission of plasmid DNA sequences injected into fertilized zebrafish eggs. Proc Natl Acad Sci U S A. 1991;88(18):7953–7957. doi:10.1073/PNAS.88.18.7953. 1910170 PMC52423

[iyad032-B13] Dahdul WM , Cui H, Mabee PM, Mungall CJ, Osumi-Sutherland D, Walls RL, Haendel MA. Nose to tail, roots to shoots: spatial descriptors for phenotypic diversity in the biological spatial ontology. J Biomed Semantics. 2014;5:34. doi:10.1186/2041-1480-5-34. 25140222 PMC4137724

[iyad032-B14] Driever W , Solnica-Krezel L, Schier AF, Neuhauss SCF, Malicki J, Stemple DL, Stainier DYR, Zwartkruis F, Abdelilah S, Rangini Z, et al A genetic screen for mutations affecting embryogenesis in zebrafish. Development 1996;123(1):37–46. doi:10.1242/DEV.123.1.37. 9007227

[iyad032-B15] Ekker SC , Larson JD. Morphant technology in model developmental systems. Genesis 2001;30(3):89–93. doi:10.1002/GENE.1038. 11477681

[iyad032-B16] Endo Y , Groom L, Celik A, Kraeva N, Lee CS, Jung SY, Gardner L, Shaw MA, Hamilton SL, Hopkins PM, et al Variants in ASPH cause exertional heat illness and are associated with malignant hyperthermia susceptibility. Nat Commun. 2022;13(1):3403. doi:10.1038/s41467-022-31088-8. 35697689 PMC9192596

[iyad032-B17] Federhen S . The NCBI taxonomy database. Nucleic Acids Res. 2012;40(D1):D136–D143. doi:10.1093/NAR/GKR1178. 22139910 PMC3245000

[iyad032-B18] Gkoutos GV , Green ECJ, Mallon AM, Hancock JM, Davidson D. Using ontologies to describe mouse phenotypes. Genome Biol. 2005;6(1):R8. doi:10.1186/gb-2004-6-1-r8. 15642100 PMC549069

[iyad032-B19] Golling G , Amsterdam A, Sun Z, Antonelli M, Maldonado E, Chen W, Burgess S, Haldi M, Artzt K, Farrington S, et al Insertional mutagenesis in zebrafish rapidly identifies genes essential for early vertebrate development. Nat Genet. 2002;31(2):135–140. doi:10.1038/ng896. 12006978

[iyad032-B20] Haffter P , Granato M, Brand M, Mullins MC, Hammerschmidt M, Kane DA, Odenthal J, Van Eeden FJM, Jiang YJ, Heisenberg CP, et al The identification of genes with unique and essential functions in the development of the zebrafish, *Danio rerio*. Development 1996;123(1):1–36. doi:10.1242/DEV.123.1.1. 9007226

[iyad032-B21] Hastings J , Owen G, Dekker A, Ennis M, Kale N, Muthukrishnan V, Turner S, Swainston N, Mendes P, Steinbeck C. ChEBI in 2016: improved services and an expanding collection of metabolites. Nucleic Acids Res. 2016;44(D1):D1214–D1219. doi:10.1093/nar/gkv1031. 26467479 PMC4702775

[iyad032-B22] Hin N , Newman M, Kaslin J, Douek AM, Lumsden A, Nik SHM, Dong Y, Zhou XF, Manucat-Tan NB, Ludington A, et al Accelerated brain aging towards transcriptional inversion in a zebrafish model of the K115fs mutation of human PSEN2. PLoS One. 2020;15(1):e0227258. doi:10.1371/JOURNAL.PONE.0227258. 31978074 PMC6980398

[iyad032-B23] Howe DG , Bradford YM, Conlin T, Eagle AE, Fashena D, Frazer K, Knight J, Mani P, Martin R, Moxon SAT, et al ZFIN, the zebrafish model organism database: increased support for mutants and transgenics. Nucleic Acids Res. 2013;41(D1):D854–D860. doi:10.1093/nar/gks938. 23074187 PMC3531097

[iyad032-B24] Howe DG , Bradford YM, Eagle A, Fashena D, Frazer K, Kalita P, Mani P, Martin R, Moxon ST, Paddock H, et al The zebrafish model organism database: new support for human disease models, mutation details, gene expression phenotypes and searching. Nucleic Acids Res. 2017;45(D1):D758–D768. doi:10.1093/nar/gkw1116. 27899582 PMC5210580

[iyad032-B25] Howe K , Clark MD, Torroja CF, Torrance J, Berthelot C, Muffato M, Collins JE, Humphray S, Mclaren K, Matthews L, et al The zebrafish reference genome sequence and its relationship to the human genome. Nature 2013;496(7446):498–503. doi:10.1038/nature12111. 23594743 PMC3703927

[iyad032-B26] Jao LE , Wente SR, Chen W. Efficient multiplex biallelic zebrafish genome editing using a CRISPR nuclease system. Proc Natl Acad Sci U S A. 2013;110(34):13904–13909. doi:10.1073/PNAS.1308335110/-/DCSUPPLEMENTAL. 23918387 PMC3752207

[iyad032-B27] Kaufman CK , White RM, Zon L. Chemical genetic screening in the zebrafish embryo. Nat Protoc. 2009;4(10):1422–1432. doi:10.1038/NPROT.2009.144. 19745824 PMC2943144

[iyad032-B28] Kawahara G , Karpf JA, Myers JA, Alexander MS, Guyone JR, Kunkel LM. Drug screening in a zebrafish model of Duchenne muscular dystrophy. Proc Natl Acad Sci U S A. 2011;108(13):5331–5336. doi:10.1073/PNAS.1102116108. 21402949 PMC3069215

[iyad032-B29] Kimelman D , Smith NL, Lai JKH, Stainier DYR. Regulation of posterior body and epidermal morphogenesis in zebrafish by localized Yap1 and Wwtr1. Elife 2017;6::e31065. doi:10.7554/ELIFE.31065. 29283341 PMC5773182

[iyad032-B30] Lawson ND , Weinstein BM. In vivo imaging of embryonic vascular development using transgenic zebrafish. Dev Biol. 2002;248(2):307–318. doi:10.1006/DBIO.2002.0711. 12167406

[iyad032-B31] Liu L , Fei F, Zhang R, Wu F, Yang Q, Wang F, Sun S, Zhao H, Li Q, Wang L, et al Combinatorial genetic replenishments in myocardial and outflow tract tissues restore heart function in tnnt2 mutant zebrafish. Biol Open. 2019;8(12):bio046474. doi:10.1242/BIO.046474. 31796423 PMC6918781

[iyad032-B32] Majczenko K , Davidson AE, Camelo-Piragua S, Agrawal PB, Manfready RA, Li X, Joshi S, Xu J, Peng W, Beggs AH, et al Dominant mutation of CCDC78 in a unique congenital myopathy with prominent internal nuclei and atypical cores. Am J Hum Genet. 2012;91(2):365–371. doi:10.1016/J.AJHG.2012.06.012. 22818856 PMC3415545

[iyad032-B33] Moens CB , Donn TM, Wolf-Saxon ER, Ma TP. Reverse genetics in zebrafish by TILLING. Briefings Funct Genomics Proteomics. 2008;7(6):454. doi:10.1093/BFGP/ELN046. PMC2899843 19028802

[iyad032-B34] Nadendla S , Jackson R, Munro J, Quaglia F, Mészáros B, Olley D, Hobbs ET, Goralski SM, Chibucos M, Mungall CJ, et al ECO: the evidence and conclusion ontology, an update for 2022. Nucleic Acids Res. 2022;50(D1):D1515–D1521. doi:10.1093/NAR/GKAB1025. 34986598 PMC8728134

[iyad032-B35] Nasevicius A , Ekker SC. Effective targeted gene “knockdown” in zebrafish. Nat Genet. 2000;26(2):216–220. doi:10.1038/79951. 11017081

[iyad032-B36] Padanad MS , Bhat N, Guo BW, Riley BB. Conditions that influence the response to Fgf during otic placode induction. Dev Biol. 2012;364(1):1–10. doi:10.1016/J.YDBIO.2012.01.022. 22327005 PMC4709014

[iyad032-B37] Postlethwait JH , Woods IG, Ngo-Hazelett P, Yan YL, Kelly PD, Chu F, Huang H, Hill-Force A, Talbot WS. Zebrafish comparative genomics and the origins of vertebrate chromosomes. Genome Res. 2000;10(12):1890–1902. doi:10.1101/GR.164800. 11116085

[iyad032-B38] Robu ME , Larson JD, Nasevicius A, Beiraghi S, Brenner C, Farber SA, Ekker SC. P53 activation by knockdown technologies. PLoS Genet. 2007;3(5):e78. doi:10.1371/JOURNAL.PGEN.0030078. 17530925 PMC1877875

[iyad032-B39] Ruzicka L , Bradford YM, Frazer K, Howe DG, Paddock H, Ramachandran S, Singer A, Toro S, Van Slyke CE, Eagle AE, et al ZFIN, the zebrafish model organism database: updates and new directions. Genesis 2015;53(8):498–509. doi:10.1002/dvg.22868. 26097180 PMC4545674

[iyad032-B40] Sabaawy HE , Azuma M, Embree LJ, Tsai H-J, Starost MF, Hickstein DD. TEL-AML1 transgenic zebrafish model of precursor B cell acute lymphoblastic leukemia. Proc Natl Acad Sci U S A. 2006;103(41):15166–15171. doi:10.1073/pnas.0603349103. 17015828 PMC1622794

[iyad032-B41] Schriml LM , Mitraka E, Munro J, Tauber B, Schor M, Nickle L, Felix V, Jeng L, Bearer C, Lichenstein R, et al Human Disease Ontology 2018 update: classification, content and workflow expansion. Nucleic Acids Res. 2019;47(D1):D955–D962. doi:10.1093/nar/gky1032. 30407550 PMC6323977

[iyad032-B42] Smith KA , Joziasse IC, Chocron S, Van Dinther M, Guryev V, Verhoeven MC, Rehmann H, Van Der Smagt JJ, Doevendans PA, Cuppen E, et al Dominant-negative alk2 allele associates with congenital heart defects. Circulation 2009;119(24):3062–3069. doi:10.1161/CIRCULATIONAHA.108.843714/FORMAT/EPUB. 19506109

[iyad032-B43] Smith JR , Park CA, Nigam R, Laulederkind SJF, Hayman GT, Wang SJ, Lowry TF, Petri V, De PJ, Tutaj M, et al The clinical measurement, measurement method and experimental condition ontologies: expansion, improvements and new applications. J Biomed Semantics. 2013;4(1):26. doi:10.1186/2041-1480-4-26. 24103152 PMC3882879

[iyad032-B44] Sprague J , Bayraktaroglu L, Bradford Y, Conlin T, Dunn N, Fashena D, Frazer K, Haendel M, Howe DGDG, Knight J, et al The zebrafish information network: the zebrafish model organism database provides expanded support for genotypes and phenotypes. Nucleic Acids Res. 2008;36(Database):D768–D772. doi:10.1093/nar/gkm956. 17991680 PMC2238839

[iyad032-B45] Sprague J , Bayraktaroglu L, Clements D, Conlin T, Fashena D, Frazer K, Haendel MA, Howe DG, Mani P, Ramachandran S, et al The zebrafish information network: the zebrafish model organism database. Nucleic Acids Res. 2006;34(90001):D581–D585. doi:10.1093/nar/gkj086. 16381936 PMC1347449

[iyad032-B46] Stuart GW , Vielkind JR, McMurray JV, Westerfield M. Stable lines of transgenic zebrafish exhibit reproducible patterns of transgene expression. Development 1990;109(3):577–584. doi:10.1242/DEV.109.3.577. 2401211

[iyad032-B47] Van Slyke CE , Bradford YM, Westerfield M, Haendel MA. The zebrafish anatomy and stage ontologies: representing the anatomy and development of *Danio rerio*. J Biomed Semant. 2014;5(1):12. doi:10.1186/2041-1480-5-12. PMC3944782 24568621

[iyad032-B48] Varshney GK , Lu J, Gildea DE, Huang H, Pei W, Yang Z, Huang SC, Schoenfeld D, Pho NH, Casero D, et al A large-scale zebrafish gene knockout resource for the genome-wide study of gene function. Genome Res. 2013;23(4):727–735. doi:10.1101/GR.151464.112. 23382537 PMC3613589

[iyad032-B49] Viktorin G , Chiuchitu C, Rissler M, Varga ZM, Westerfield M. Emx3 is required for the differentiation of dorsal telencephalic neurons. Dev Dyn. 2009;238(8):1984–1998. doi:10.1002/DVDY.22031. 19650145 PMC2975037

[iyad032-B50] Westerfield M . The Zebrafish Book: a Guide for the Laboratory use of Zebrafish (Danio rerio). 4th ed. Eugene (OR): University of Oregon Press; 2000.

[iyad032-B51] Wheeler MA , Jaronen M, Covacu R, Zandee SEJ, Scalisi G, Rothhammer V, Tjon EC, Chao CC, Kenison JE, Blain M, et al Environmental control of astrocyte pathogenic activities in CNS inflammation. Cell 2019;176(3):581–596.e18. doi:10.1016/J.CELL.2018.12.012. 30661753 PMC6440749

[iyad032-B52] Widrick JJ , Alexander MS, Sanchez B, Gibbs DE, Kawahara G, Beggs AH, Kunkel LM. Muscle dysfunction in a zebrafish model of Duchenne muscular dystrophy. Physiol Genomics. 2016;48(11):850–860. doi:10.1152/PHYSIOLGENOMICS.00088.2016. 27764767 PMC6223571

[iyad032-B53] Williams TD , Mirbahai L, Chipman JK. The toxicological application of transcriptomics and epigenomics in zebrafish and other teleosts. Brief Funct Genomics. 2014;13(2):157–171. doi:10.1093/BFGP/ELT053. 24397978

[iyad032-B54] Yu D , Zhang P, Li J, Liu T, Zhang Y, Wang Q, Zhang J, Lu X, Fan X. Neuroprotective effects of *Ginkgo biloba* dropping pills in Parkinson's Disease. J Pharm Anal. 2021;11(2):220–231. doi:10.1016/J.JPHA.2020.06.002. 34012698 PMC8116202

[iyad032-B55] Zhang J , Wang C, Shen Y, Chen N, Wang L, Liang L, Guo T, Yin X, Ma Z, Zhang B, et al A mutation in ADIPOR1 causes nonsyndromic autosomal dominant retinitis pigmentosa. Hum Genet. 2016;135(12):1375–1387. doi:10.1007/s00439-016-1730-2. 27655171

[iyad032-B56] Zon LI , Peterson RT. In vivo drug discovery in the zebrafish. Nat Rev Drug Discov. 2005;4(1):35–44. doi:10.1038/nrd1606. 15688071

[iyad032-B57] Zu Y , Tong X, Wang Z, Liu D, Pan R, Li Z, Hu Y, Luo Z, Huang P, Wu Q, et al TALEN-mediated precise genome modification by homologous recombination in zebrafish. Nat Methods. 2013;10(4):329–331. doi:10.1038/nmeth.2374. 23435258

